# Identification of JUN gene and cellular microenvironment in response to PD-1 blockade treatment in lung cancer patients via single-cell RNA sequencing

**DOI:** 10.18632/aging.205932

**Published:** 2024-06-13

**Authors:** Yuxuan Wang, Tao Ran, Yunke Li, Lei Tian, Lifeng Yang, Zhidong Liu, Biao Yao

**Affiliations:** 1 No.2 Department of Thoracic Surgery, Beijing Tuberculosis and Thoracic Tumor Research Institute/Beijing Chest Hospital, Capital Medical University, Beijing, China; 2 Department of Oncology, Tongren People’s Hospital, Tongren, Guizhou, China; 3 Beijing Digitf Biotechnology Co., Ltd, Beijing, China

**Keywords:** PD-1, single-cell sequencing, cancer immunotherapy, JUN, micro-environment

## Abstract

Exploring the molecular mechanisms of PD-1/PDL-1 blockade for non-small cell lung cancer (NSCLC) would facilitate understanding for tumor microenvironment (TME) and development of individualized medicine. To date, biomarkers of response to PD-1 blockade therapy were still limited. In this study, we hypothesize that cell type in the tumor microenvironment can influence the effect of PD-1 blockade immunotherapy through specific genes. Therefore, we re-analyze the single-cell RNA sequencing data and validation in tissue from lung adenocarcinoma patients. Dynamic changes of cellular subpopulation were observed after anti-PD-1 immunotherapy among TMEs between primary/metastasis or good/poor response patients. Non-exhausted CD8 T cells and dysregulated genes were observed in responsing patients from PD-1 blockade therapy. Among all changed genes, JUN, involved in PD-1 blockade immunotherapy pathway, and could be considered as a PD-1 responsing biomarker.

## INTRODUCTION

Although the morbidity and mortality of lung cancer was reported to be declined recently, especially non-small cell lung cancer (NSCLC), they were still higher than other cancer types [1].

Immune-checkpoint blockade (ICB), targeting PD-1 or PD-L1, exhibited exciting antitumor capabilities, and greatly improved prognosis for patients in multiple tumors [2–4]. However, only around 30% of the lung cancer patients could benefit from PD-1 blockade treatment [5], and differences response from individuals were also observed in NSCLC patients [6]. So, investigation of cellular and molecular mechanisms of response for PD-1 inhibitor would enable us to understand the prognosis better and develop appropriate strategy for individual immunotherapy.

Although traditional bulk RNA sequencing reported multiple biomarkers (such as EGFR, ERBB2, and BRAF, etc.) for lung cancer, it encountered great challenges in illustrating gene regulation in cellular subtypes, such as CD4 regulation T cells, or exhausted CD8+ T cell which played crucial roles in tumor progress [7, 8]. Single-cell RNA sequencing enabled us to in-depth characterize of cellular subtype in TME, for example, M1/M2 macrophage ratio influencing the response rate for anti-PD-1/PD-L1 therapy [6], exhausted CD8+ T cells enriched in cancer tissues [9], etc.

T cell exhaustion is characterized by the gradual decline of effector function (reduction in IL-2, TNF-α, and IFN-γ production) and the continued presence of inhibitory receptors such as PD-1, Tim-3, CTLA-4, lymphocyte-activation gene 3 (LAG-3), and CD160, accompanied by a distinct transcriptional profile separate from that of functional effector or memory T cells [10]. Terms such as tolerance, anergy, and exhaustion are utilized to depict T cells with diminished responsiveness. Tolerance denotes the primary mechanism to forestall autoimmunity through the central or peripheral inactivation of self-reactive T cells [11, 12]. Anergy characterizes T cells that are incompletely activated due to the absence of co-stimulatory signals and/or significant co-inhibitory stimulation [13, 14]. Exhaustion, among these terms, specifically delineates a functional yet hyporesponsive state resulting from initial activation in the context of chronic infection or tumor, distinguishing it from tolerance and anergy.

Previous study demonstrated that αPD-L1 treatment leads to interleukin-7 receptor (IL-7R) (CD127) expression on exhausted T cells during chronic lymphocytic choriomeningitis virus (LCMV) infection which could make exhausted CD8+ T cells responsive to IL-7, a key cytokine known for promoting long-term survival of mature effector CD8+ T cells by upregulating the anti-apoptotic marker Bcl-2 and the generation of memory T cell phenotype [15]. Further study found that treatment with IL-7 alone did not significantly alter the course of chronic LCMV infection, although it was crucial for the homeostatic proliferation of memory CD8+ T cells. However, combined therapy using αPD-L1 and IL-7 showed additive effects, leading to the expansion of LCMV-specific CD8+ T cells producing both IFN-γ and tumor necrosis factor-alpha (TNF-α). IL-7 appears to collaborate with IL-15 to maintain Bcl-2 expression, essential for signal transducer and activator of transcription 5 (STAT-5) phosphorylation and the generation of long-lived effector CD8+ T cells [16]. While αPD-L1 treatment increases STAT-5 phosphorylation in exhausted CD8+ T cells upon additional IL-7 stimulation, no difference was observed in the expression of the IL-15 receptor CD122 between treated and untreated exhausted CD8+ T cells in chronically LCMV-infected hosts. This disparity in homeostatic cytokine receptor expression patterns may contribute to only partially improved survival of αPD-L1 treated exhausted CD8+ T cells, even in an antigen-free environment. These findings highlight significant changes occurring possibly at both transcriptional and epigenetic levels in exhausted CD8+ T cells, which are not adequately altered by αPD-L1 treatment [15].

c-Jun amino-terminal kinase (c-Jun), p38MAPK and ERK are three parallel pathways involved in the MAPK pathway [17]. Recent studies focused on the association between the PD-1/PD-L1 axis and the MAPK pathway. Stutvoet et al. found that inhibition of the MAPK pathway prevented epidermal growth factor and IFN-γ-induced CD274 mRNA and PD-L1 protein and membrane upregulation in lung adenocarcinoma cells [18]. Jalali et al. revealed p-P38 and p-ERK were decreased in all HL lines after using an anti-PD-L1 antibody [19].

In this study, we hypothesized that differences in the response of the PD-1 inhibitor among lung cancer patients was related to key genes and lymphocytes paracrine activation, and reanalyzed the PD-1 blockade responsing associated single-cell RNA sequencing (scRNAseq) data shared by Professor Zemin Zhang to investigate the cellular and molecular mechanisms for PD-1 blockades.

## MATERIALS AND METHODS

### Data process and tissue specimens

The scRNAseq data of eight patients were collected from GSE179994 [20]. The data comprised five male and three female patients ranging in age from 48 to 73 years (all: 60±8.8; male: 57.8±9.7; female: 63.7±8.3) with a clinical diagnosis of LUAD. All patients were treated with the same strategy (pembrolizumab + carboplatin + pemetrexed). The data were divided into groups based on the selected sample data included biopsy site (such as lung or lymph node metastasis), response to therapy (good or poor), and all data were performed quality control (QC) (Table 1). The metastasis data from PD-1 blockade good responsing patients were defined as Apre group (before treatment) and Apost group (after treatment); the metastasis data from PD-1 blockade poor responsing patients were defined as Bpre group (before treatment) and Bpost group (after treatment); the primary data from PD-1 blockade good responsing patients were defined as Cpre group (before treatment) and Cpost group (after treatment).

**Table 1:** Sample data information and key group markers in single cell RNAseq.

**Sample ID**	**Patient ID**	**PD-1 Inhibitor response**	**Biopsy site**	**Timepoint**
A01_ut_meta	P010	Yes	LN metastasis	A01pre
A02_ut_meta	P019	Yes	LN metastasis	A02pre
A01_tr_meta	P010	Yes	LN metastasis	A01post
A02_tr_meta	P019	Yes	LN metastasis	A02post
B01_ut_meta	P001	Yes	LN metastasis	B01pre
B02_ut_meta	P013	Yes	Liver metastasis	B02pre
B02_tr_meta	P001	No	Right lung tumor	B02post
B02_tr_meta	P013	No	LN metastasis	B02post
C01_ut_pri	P029	Yes	Left lung tumor	C01pre
C02_ut_pri	P030	Yes	Right lung tumor	C02pre
C03_ut_pri	P033	Yes	Right lung tumor	C03pre
C04_ut_pri	P035	Yes	Right lung tumor	C04pre
C01_tr_pri	P029	Yes	Left lung tumor	C01post
C02_tr_pri	P030	Yes	Right lung tumor	C02post
C03_tr_pri	P033	Yes	Right lung tumor	C03post
C04_tr_pri	P035	Yes	Right lung tumor	C04post

This study was approved by the Center Ethics Committee of Beijing Chest Hospital (JYS-2021-011). Signed informed consents were collected from all participants. 22 biopsies from LUAD patients were collected and stored at -80° C in RNAlater (Thermo Fisher Scientific, USA).

### scRNAseq data processing and clustering

The human reference genome (vGRCh38-3.0.0) was downloaded from the 10X Genomics website in February 2022 (https://cf.10xgenomics.com/supp/cell-exp/refdata-gex-GRCh38-2020-A.tar.gz). A raw and filtered RNA-expression matrix was generated by Kallisto/bustools (kb, v0.24.4) pipeline and mapped to the reference genome. Single-cell counts of each sample were generated by kb to count. Then, the features of the filtered, barcode, and matrix files were analyzed using an scRNAseq AnaSys™ platform (Digitf bioctech, Beijing, China), which was integrated by Python (v3.8.10), anndata (ad) (v0.7.6), and scanpy (sc) (v1.7.2) packages. For further analysis, the cells and genes of the sample data were filtered using the sc.pp.filter_cells and sc.pp.filter_genes functions of the scRNAseq AnaSys^™^ platform.

To reduce the technical defects of the capture of low-quality cells, doublets cells, etc., cells were filtered based on the following criteria: 1) < 1,000 or > 25,000 unique molecular identifiers (UMIs, representing unique mRNA transcripts); 2) < 500 or > 5,000 genes in each sample; or (3) > 10% UMIs derived from mitochondrial genes. In the scRNAseq AnaSys™ platform, scanpy’s external module Scrunlet was used to identify potential doublet cells using default parameters [21, 22]. Cells were labeled on predicted results and filtered out. After implementing QC procedures, the gene expression matrix was normalized using tools in the scRNAseq AnaSys™ platform. Subsequently, the normalized counts were natural logarithm transformed (X = Log (X + 1)) using the sc.pp.log1p function, which is integrated into the scRNAseq AnaSys™ platform. The log-transformed expression values of each sample were used for downstream analysis.

Briefly, the clustering analysis of cell types and subtypes was composed of three steps. The first step (Louvain resolution = 2.0) was performed on all cells and identified 13 clusters for the subtype cells, including C1 (cluster)_CD4 naïve T cells (marker: CCR7), C2_CD4 central memory T cells (Tcm) (markers: ANXA1, LMNA, MYADM and RGCC), C3_CD4 T effector memory cells (Tem) (GZMA, CCL5 and GZMK), C4_CD4 CD69 T cells (markers: FOS, FOSB and DUSP1), C5_CD4 ISG15 T cells (markers: IFI27, ISG15, IFI6 and LY6E), C6_CD4 RPL T cells (markers: RPS29, RPL41, RPS27 and TCF7), C7_CD4 Th1-like cells (markers: CXCL13, TOX, PDCD1 and IFNG), C8_CD4 Treg cells (markers: LAYN, CCR8 and FOXP3), C9_CD4 T proliferation cells (markers: MKI67, STMN1, TYMS, TUBA1B, YUBB, UBA52, CRIP1, YNFRSF9 and CD69), C10_CD4 XCL1 T cells (markers: XCL1, XCL2, MYADM, CAPG and CD69), C11_CD8 non-exhausted T cells (marker: GZMK), C12_CD8 proliferation T cells (markers: MKI67, STMN1 and ENTPD1), and C13_CD8 exhausted T cells (markers: TIGIT, HAVCR2 and ITGAE). For investigating the mechanism of anti-PD-1 therapy, the second step (with Louvain resolution = 2.0) was performed on CD4 T cells and CD8 T cells separately to divide these cells into subsets expressing the different immune cell lineages of the marker genes. The third step (with Louvain resolution = 2.0) was performed on CD8 T cell clusters to further divide cells into subclusters, such as non-exhausted CD8 T cells (CD8 T non-exhaust), exhausted CD8 T cells (CD8 Tex) and proliferation CD8 T cells (CD8 T prolif).

### LUAD RNA data from TCGA database and Kaplan-Meier plotter (KM) analysis

Clinical information and transcript per million (TPM) data of lung cancer in TCGA database were downloaded from the UCSC Xena Data center (https://xenabrowser.net/datapages/). The Molecular Signatures Database (MSigDB) was downloaded from its official website (https://www.gsea-msigdb.org/gsea/msigdb/index.jsp). Gene Set Enrichment Analysis (GSEA) was performed by clusterProfiler (https://github.com/YuLab-SMU/clusterProfiler). Kaplan-Meier (KM) plotter (https://kmplot.com/) was utilized to explore the prognosis of potential key genes.

### Bulk-tissue RNA sequencing and data analysis

The total RNA of samples was extracted separately from 200~400 mg of homogenized biopsy tissue using an RNAsimple Kit (TIANGEN, Cat# DP419, China) following the manufacturer’s instructions. RNA integrity number (RIN) validation was tested using an Agilent RNA 6000 Nano Kit (Agilent, Cat#5067-1511, USA). The RIN values for 22 tissue biopsies ranged from 7.2 to 8.3 (median: 7.8). To prepare RNA sequencing libraries, the total RNA was further purified using an RNAclean Kit (Tiangen, Cat# DP210831, China) according to the manufacturer’s instructions. High-quality DNA-free RNA was used for rRNA depletion (TIANSeq rRNA depletion kit, Cat#NR101, China) and library preparation with cDNA synthesis (TIANSeq Fast RNA Library Kit, Cat#NR102, China) following the manufacturers’ instructions. RNA sequencing and quality control were performed using Illumina HiSeq 2500 sequencing platforms (Illumina, USA).

Initial RNA sequencing data analysis and preparation were conducted via the RNA sequencing analysis pipeline platform v1.0 (Digitf Biotech, Beijing, China). This pipeline included fast QC software for quality control and reads filtering for adaptor and rRNA and mtDNA sequences using Python scripts (v1.2). All clean reads were aligned with the human reference genome (vGRCh38-3.0.0). Transcript quantification and gene expression (raw read counts) were conducted with Cufflinks (v2.0) to compare reference annotations (measured as Transcripts Per Million, TPM).

### Immunohistochemistry

IHCs were conducted by the Envision two-step method. After paraffin sectioning, the sections were deparaffinized using conventional xylene and ethanol, followed by three washes with PBS, each lasting 3 minutes. Subsequently, the sections were incubated in a dark environment with 3% hydrogen peroxide for 10 minutes to block endogenous peroxidase activity. The sections were then incubated with primary and secondary antibodies. Diaminobenzidine (DAB) staining was performed on the sections, followed by counterstaining with hematoxylin. After gradient dehydration in different concentrations of ethanol, the sections were observed under a microscope. The antigen used was rabbit polyclonal antibody JUN [9165T, CST, US]. We selected 22 sample slices of tissue from clinical cases at Tongren People’s Hospital in Guizhou based on disease progression and recurrence, and utilized the c-Jun monoclonal antibody (9165S, Cell Signaling Technology, USA) for staining analysis.

### Statistics

Statistical analysis of all data was performed using R (v4.0.2) and Python (v3.79) software. Log-rank tests were conducted using KM survival analysis. Student’s t-tests were conducted for normalized distributed data, Mann-Whitney test was used for abnormal distributed data. All figures are marked by distinctive symbols indicating statistical significance (ns: P>0.05; *: P≤0.05; **: P≤0.01; ***: P≤0.001; ****: P≤0.0001).

### Software and algorithms

All software and algorithms were included in the scRNAseq AnaSys™ platform. Sources and identifiers are as follows:

- Annadata, pypi, https://github.com/theislab/anndata
- CellRanger v6.1.0,10x Genomics, http://10xgenomics.com
- Ggplot2, bioconductor, https://ggplot2.tidyverse.org
- Ggpubr bioconductor, https://github.com/kassambara/ggpubr
- Gseapy v0.10.7, pypi, https://pypi.org/project/gseapy
- Harmonypy, pypi, https://github.com/slowkow/harmonypy
- Kallistobustools v0.24.4, pypi, https://github.com/pachterlab/kb_python
- Scanpy v1.7.2, bioconda, https://github.com/theislab/scanpy
- Scirpy v0.7.0, bioconda, https://github.com/icbi-lab/scirpy
- Scrublet v0.2.3, pypi, https://github.com/swolock/scrublet
- Statannot, pypi, https://pypi.org/project/statannot

### Data availability

All datasets analyzed for this study can be found in the Gene Expression Omnibus (GEO) under accession code GSE179994 (scRNAseq).

## RESULTS

### Clinical features and cellular immune micro-environment of LUAD patients

**Figure 1:**
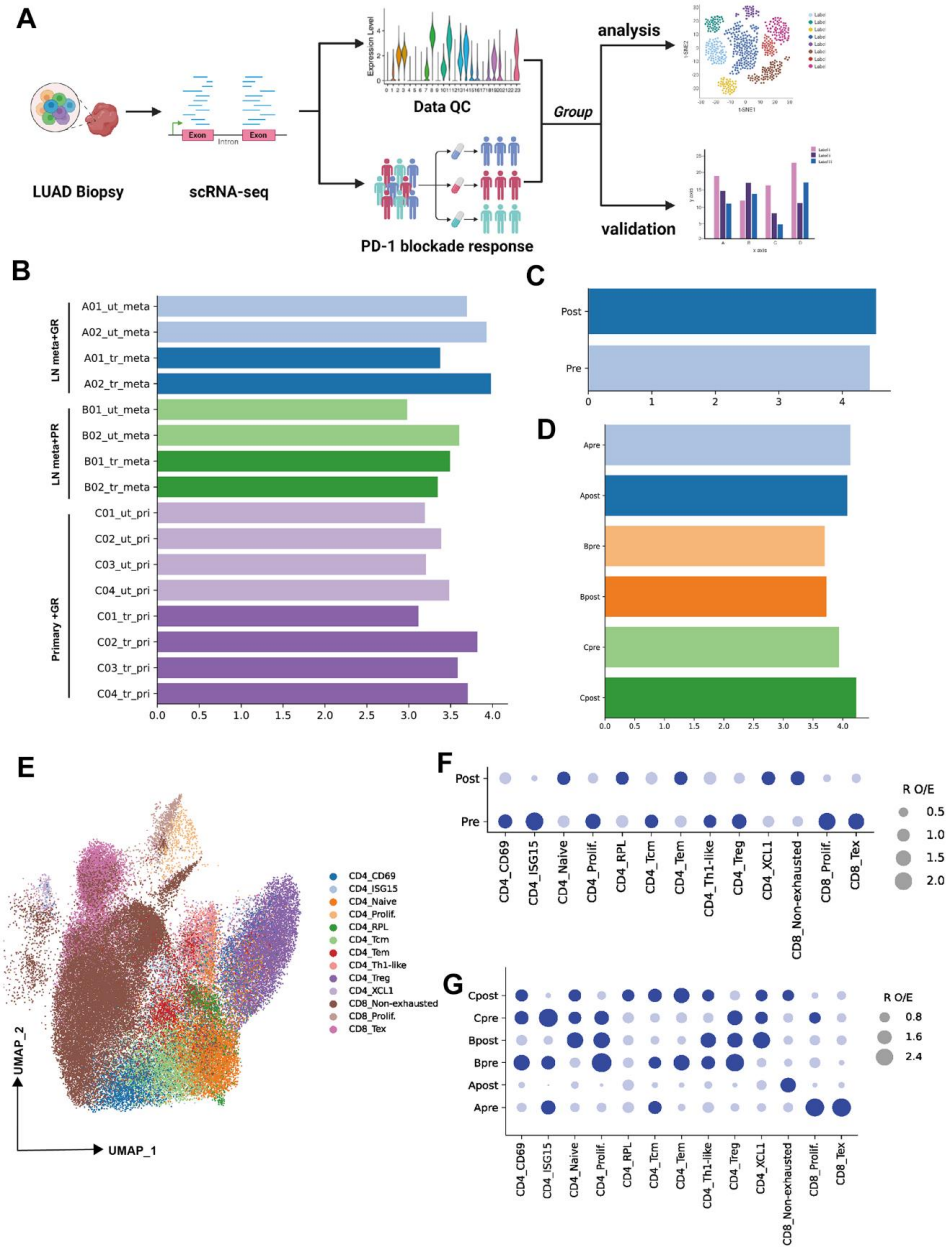
**Single-cell transcriptional analysis of T lymphocytes from LUAD patient biopsies.** (**A**) Schematic showing the analysis of procedures used for this study (created using BioRender.com). (**B**) Bar plot showing the cell number (Log_10_ transformed) of samples selected from primary patients’ data (ut: untreatment; tr: treatment; meta: lymph node or liver metastasis; pri: primary tumor). (**C**) Bar plot showing the cell number (Log_10_ transformed) of pre-PD-1 blockade therapy and post-PD-1 blockade therapy. (**D**) Bar plot showing the cell number (Log_10_ transformed) of groups which were defined by biopsy sites (metastasis or primary tumor) and response of PD-1 blockade therapy (good or poor response). (**E**) Two-dimensional UMAP plot of single-cell RNA-Seq (scRNA-Seq) performed on groups A, B, and C after PD-1 blockade therapy (horizontal axis: UMAP_1, vertical axis: UMAP_2). (**F**) Patient group preference for each CD4 and CDD8 subcluster measured using the RO/E index (dark blue: enrichment, light blue: depletion).

Based on our inclusion criteria, such as the quality of single-cell sequencing data, response of PD-1 blockades combined chemotherapy treatment, primary or metastatic tumor), as mentioned in the Materials and Methods section, the study design and bioinformatics analysis flow charts were generated (Figure 1A). Clinical characteristics and response of PD-1 blockade were summarized (Table 1 and Supplementary Table 1).

To investigate the lymphocytes heterogeneity of PD-1 blockade therapy from biopsy sites, the cell count of all samples or groups were visualized (Figure 1B–1D, log_10_ transformed). Then 13 cellular subtypes from CD4 or CD8 were obtained (Unsupervised clustering by UMAP identified 13 clusters, as mentioned in the Methods section) (Figure 1E). R O/E analysis among groups (pre and post) demonstrated the change of subpopulation from CD4 or CD8 T cells. For example, the depletion of CD4 ISG15, CD4 prolif, CD4 Treg, CD8 prolif and CD8 Tex were observed in post-group, while the CD4 naïve, CD4 RPL, CD4 Tem, CD4 XCL and CD8 non-exhausted enriched in post-group (Figure 1F). To unravel the heterogeneity and complexity of cellular subtype among biopsy site groups (metastasis or primary) before (pre) and after (post) PD-1 blockade therapy, R O/E analysis was also performed for three groups (Group A, B, C). Depletion trend for CD8 Tex, CD8 prolif, CD4 ISG15, CD4 Tcm and enrichment for CD8 non-exhausted were observed in group Apost (Figure 1G). Depletion trend for CD4 Tem, CD4 Tcm, CD4 ISG15 and enrichment for CD4 naïve and CD4 XCL1 were observed in group Bpost (Figure 1G). Depletion trend for CD4 Treg, CD4 prolif, CD4 ISG15, CD8 prolif and enrichment for CD4 RPL, CD4 Tcm, CD4 Tem and CD8 non-exhausted were in group Cpost (Figure 1G).

### Characteristics of CD4+ T subtypes, cellular interaction of Treg and CD8 subclusters

**Figure 2:**
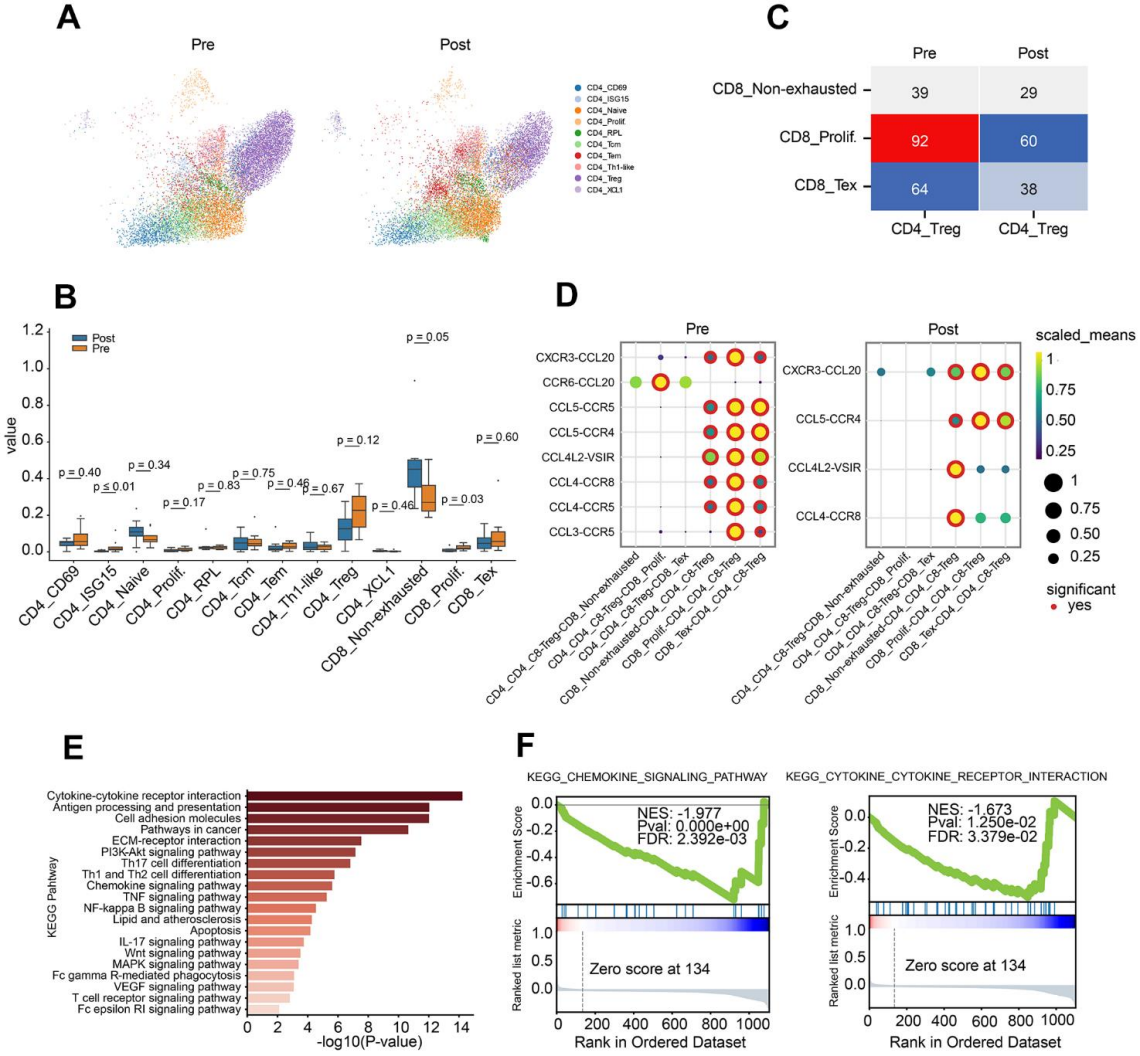
**Characteristics of CD4 T lymphocyte subclusters and cellular communication.** (**A**) Two-dimensional UMAP plot of CD4 T lymphocyte subclusters (unsupervised clustering distribution) in pre and post groups; UMAP plots are colored based on the cell subtypes of the CD4 T cells (horizontal axis: UMAP_1, vertical axis: UMAP_2). (**B**) Proportions of CD4 and CD8 cell subpopulations between pre-treatment and post-treatment using Box plots (value: cell proportion, t test). (**C**) The cellular communication of regulatory T cells (Treg) and CD8 T subclusters in pre and post groups. (**D**) The ligand–receptor pairs of chemokine receptor and receptor between Treg cells and CD8 subclusters (CD8 T non-exhausted, CD8 Tex and CD8 prolif.) using Dot plots (value: Scaled Means, CD8 T non-exhausted: non-exhausted CD8 T cells, CD8 prolif: proliferation CD8 T cells, CD8 Tex: exhausted CD8 T cells). (**E**) KEGG pathway enrichment analysis of differential gene expression of Treg cells in pre-group and post-group. (**F**) GSEA curve for chemokine signal pathway (left) and cytokine and cytokine receptor interaction (right) of Treg cells in pre-group and post-group.

To further explore the potential mechanism for PD-1 inhibitor, CD4 or CD8 T cells were extracted, and the subpopulation was identified by canonical markers. Totally, 10 subclusters of CD4 T cells were obtained in pre- or post-group. Enrichment of CD4 naïve and CD4 Tem, decrease of CD4 Treg and CD4+T prolif were observed in post-group (Figure 2A, 2B). Cellular interaction between Treg and CD8 subclusters demonstrated the decrease tread between CD8 Tex and Treg, CD8 prolif and Treg in post-group (Figure 2C).

To investigate the crosstalk between Treg and CD8 T cell sub-clusters before and after PD-1 blockade therapy, chemokine ligand-receptor pairs were analysis. The results revealed a down-regulated trend for CCR6-CCL20, CCL5-CCR5, CCL4-CCR5 and CCL3-CCR5 in pre-group (Figure 2D). To further investigate the role of Treg, differential gene analysis was performed on Treg cells between before and after PD-1 blockade therapy. Pathway analysis demonstrated the dysregulated genes involved in lots of cancer immune related pathways, such as cytokine-cytokine receptor interaction, chemokine pathway, Th1/Th2 balance pathway and pathways in cancer (Figure 2E). Besides, down-regulation of chemokine pathway and cytokine-receptor interaction were also observed in post-group (Figure 2F).

**Figure 3:**
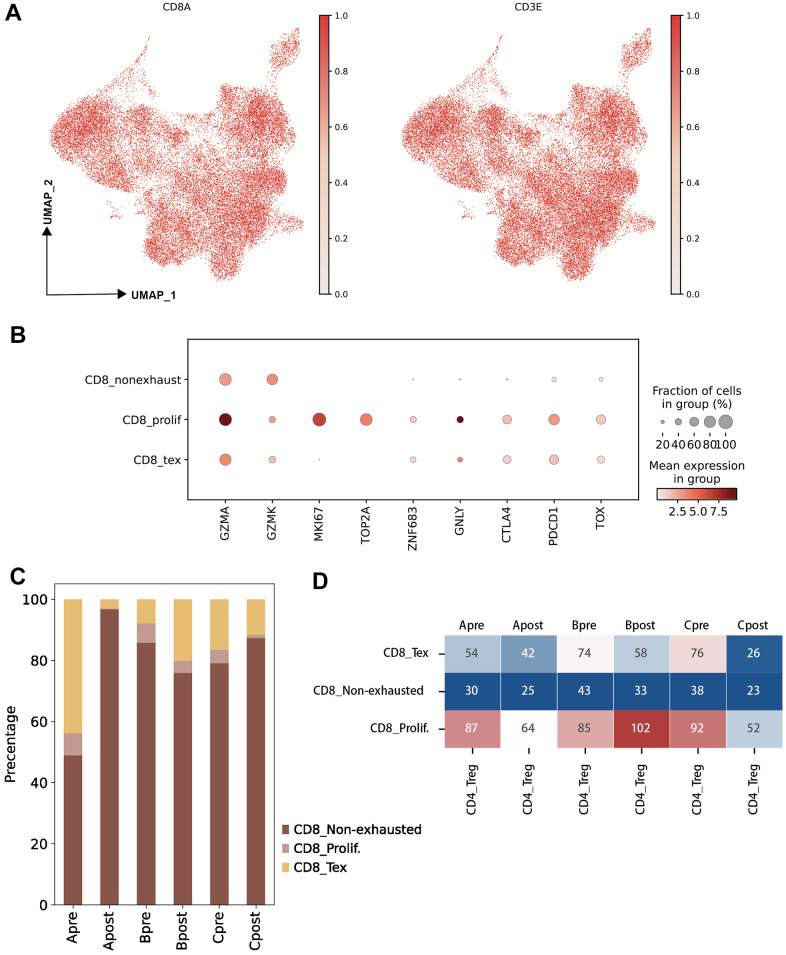
**Characteristic of CD8 T lymphocyte subclusters and identified unique cell clusters resistant to PD-1 blockade therapy.** (**A**) UMAP plots showing the CD8 T cell subcluster and majority cell types. The dot plots are colored based on CD8A and CD3E gene (left: UMAP clustering CD8 T subset, right: Lymphocytes defined by CD3E; horizontal axis: UMAP_1, vertical axis: UMAP_2). (**B**) The statistics of gene markers and cell counts in each subcluster of CD8 T cells using bubble plot (filtered by > 500 cells; CD8 T non-exhausted: non-exhausted CD8 T cells, CD8 prolif: proliferation CD8 T cells, CD8 Tex: exhausted CD8 T cells). (**C**) The percentage of CD8 T cell subclusters in each group (A: LN metastasis and good response of PD-1 blockade; B: LN metastasis and poor response of PD-1 blockade; C: Primary tumor and good response of PD-1 blockade). (**D**) Hot map showing the cellular interactions between CD4 Treg cells and CD8 T cell subclusters in each group (value: Ligand-Receptor cunts).

CD8 T cells were also extracted by canonical CD3E and CD8A markers (Figure 3A). Then, the canonical markers contributing to three major clusters of CD8 T cells (CD8 Tex, CD8 non-exhausted and CD8 prolif) were analyzed (Figure 3B). CD8 non-exhaust T cells with high expression of GZMK and GZMA indicated its killing role on tumor cells, CD8 Tex with PDCD1 and TOX suggested its loss of function, and high expression of proliferation feature gene (MKI67) and exhausted related genes (GZMA, PDCD1, TOX) on CD8 prolif suggested the dual characteristics of proliferation and exhaustion (Figure 3B and Supplementary Table 3).

### JUN is a key marker gene for PD-1 blockade

To investigate the differential of cellular on pre-group and post group, three groups were generated following biopsy site and PD-1 blockade response (Group A: LN meta plus Good; Group B: LN meta plus Poor; Group C: Pri plus Good, see “Material and Methods”). Large difference of percentage of CD 8 sub-clusters were observed between Group Apre and Apost (Figure 3C). Significantly interact between Treg and sub-clusters of CD8 Tex, CD8prolif in pre-group and post-group were obtained (Figure 3D) which indicated that Treg and CD8 Tex cells maybe potential targets on PD-1 blockade therapy.

**Figure 4:**
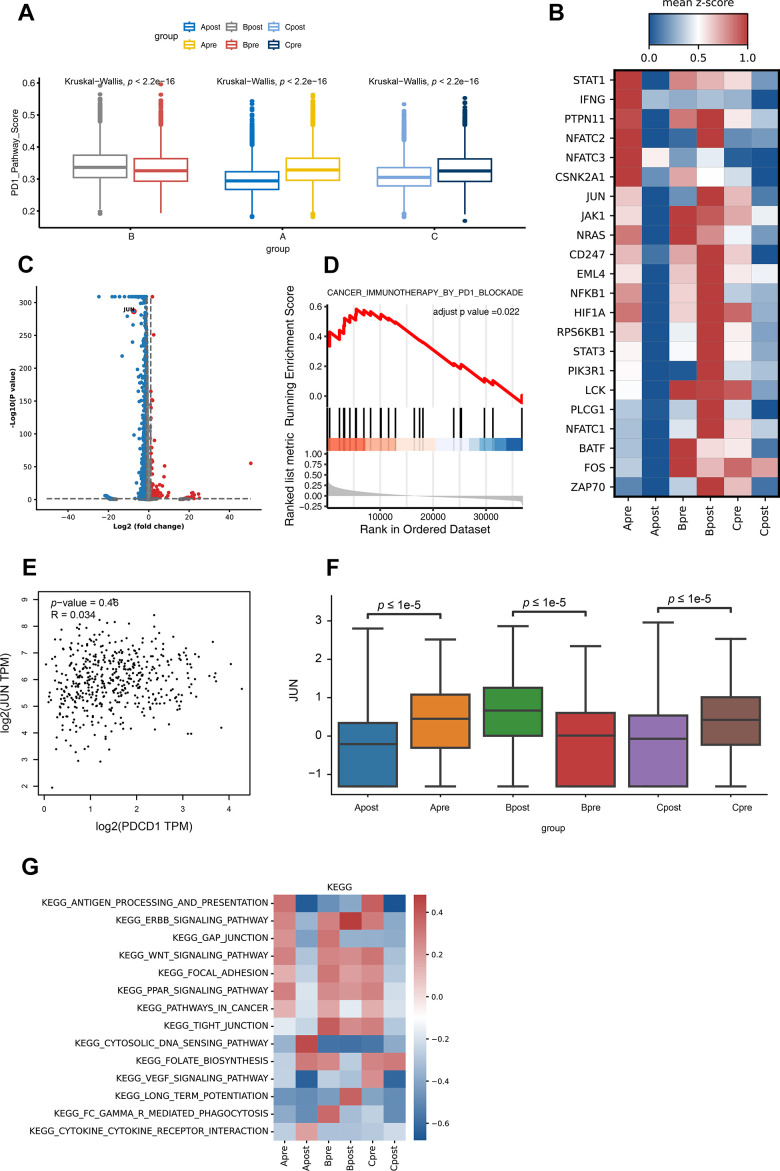
**Differential gene expression of PD-1 pathway and KEGG pathway enrichment analysis for CD8 T lymphocytes in each group.** (**A**) The PD-1 pathway score of group A, B and C in PD-1 blockade therapy pre-treatment (Apre, Bpre, Cpre) and post-treatment (Apost, Bpost, Cpost) using Box and whisker plots (value: cell proportion, t test, *:P≤0.05; **:P≤0.01; ***:P≤0.001; ****:P≤0.0001). (**B**) The hot plot showing differential genes in all groups before (pre) and during (post) the PD-1 blockade therapy (value: mean z-score; red: high expression; blue: low expression). (**C**) The JUN gene in CD8 T lymphocytes is shown by volcano plot before (pre) and during (post) the PD-1 blockade therapy (P-value < 0.05; |Log_2_FC| ≥ 1). (**D**) GSEA analysis of JUN in lung cancer expression data from TCGA. Result showed JUN positively involved in immunotherapy in PD1 blockade (adjust p-value <0.05). (**E**) The correlation expression between JUN and PDCD1 from LUAD RNA data in TCGA database. (**F**) The box plot showing the JUN expression level of CD8 T lymphocytes in group A, B and C before (pre) and during (post) the PD-1 blockade therapy. (**G**) GSVA analysis of 14 pathways for CD8 T lymphocytes in before (pre) and during (post) the PD-1 blockade therapy groups.

To explore more detailed mechanism, firstly, the PD-1 pathway score between pre-treatment and post-treatment among all groups were investigated. Interestingly, the PD-1 pathway scores down-regulated after treatment in good response group (Group A and C); and up-regulated after treatment in poor response group (Group B) (Figure 4A). Not surprisingly, lots of genes changed after treatment (Figure 4B, 4C). Among all changed genes, JUN is significantly involved in PD-1 pathway which indicated its potential role in PD-1 inhibitor treatment (Figure 4D). Then, the up-regulation of the JUN in the good response group (Group A and C) was highly correlated with the PD-1 pathway compared to that of poor response group (B) (Figure 4B, 4C, 4F; *P*<0.05). To further demonstrate that JUN is associated with PD-1 signaling pathway activation (search in KEGG database), we extracted the RNA sequencing data of lung cancer patients with LUAD from the TCGA database to analyze the correlation between JUN and PD-1 blockade treatment response. We found that the correlation was not high in patients with poor PD-1 treatment response (R=0.034, *P*=0.46, Figure 4E), suggesting that it might be not associated with PD-1 blockade treatment effect in patients. Downregulation of antigen process and presentation, ERBB, PPAR, WNT and Focal signal pathway were observed after PD-1 treatment in good response groups (A and C). In contrast, there was no significant change in group B (Figure 4G and Supplementary Table 2).

In KEGG database, JUN located down-stream of the PD-1 pathway and activated T cells indirectly, which enable T lymphocytes to secrete interleukin 2 as well as cytokines and chemokines. The high expression level of JUN gene may suggest a higher tumor burden and good response for blocking PD-1 (https://www.genome.jp).

### Validation of the JUN predictive role in effect of PD-1 blockade therapy by bulk-tissue RNA sequencing

**Figure 5:**
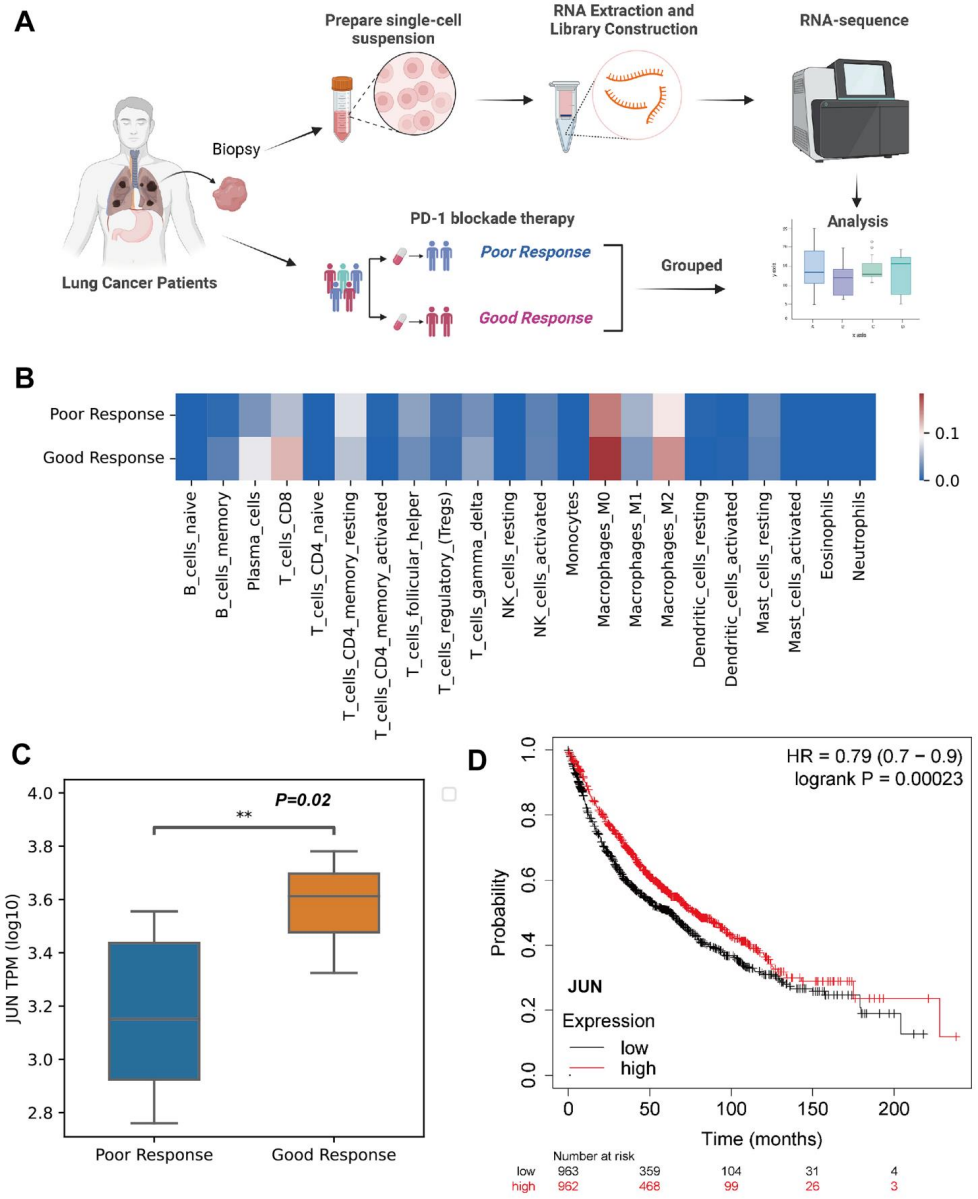
**Validation of JUN expression in the CD8 lymphocytes as an indicative biomarker of sensitivity to PD-1 blockade in lung adenocarcinoma.** (**A**) Schematic showing the RNA-seq validation of procedures used for JUN gene (created using BioRender.com). (**B**) Heat map of immune cell fractions in the microenvironment of lung cancer patients (n=17, LUAD: 3 patients, LUSC: 14 patients). (**C**) Box and whisker plot showing the JUN expression level of poor or good response for PD-1 blockade therapy in lung cancer patients using RNA sequence (TPM; Log10; good response: n = 8; poor response: n = 9; P=0.02) with non-parametric Wilcoxon test. (**D**) Kaplan-Meier plotter showing the probability of JUN in lung cancer patients (Logrank test, P=0.00023).

To validate the finding of JUN as a predictive marker in PD-1 blockade therapy, biopsy tissues from lung adenocarcinoma patients were collected and performed RNA sequencing (Figure 5A). Specific immune cell patterns in the microenvironment were observed between good and poor response (Figure 5B). Importantly, JUN was significantly high in the good response group (*P*=0.02, Figure 5C).

A larger cohort from KMplot database of lung cancer was enrolled for survival analysis which exhibited high expression of JUN resulted a better prognosis (*P*=0.00023, Figure 5D). This finding indicated that high expression of JUN may be a predictive factor of prognosis.

### Validation of the JUN predictive role by IHC

**Figure 6:**
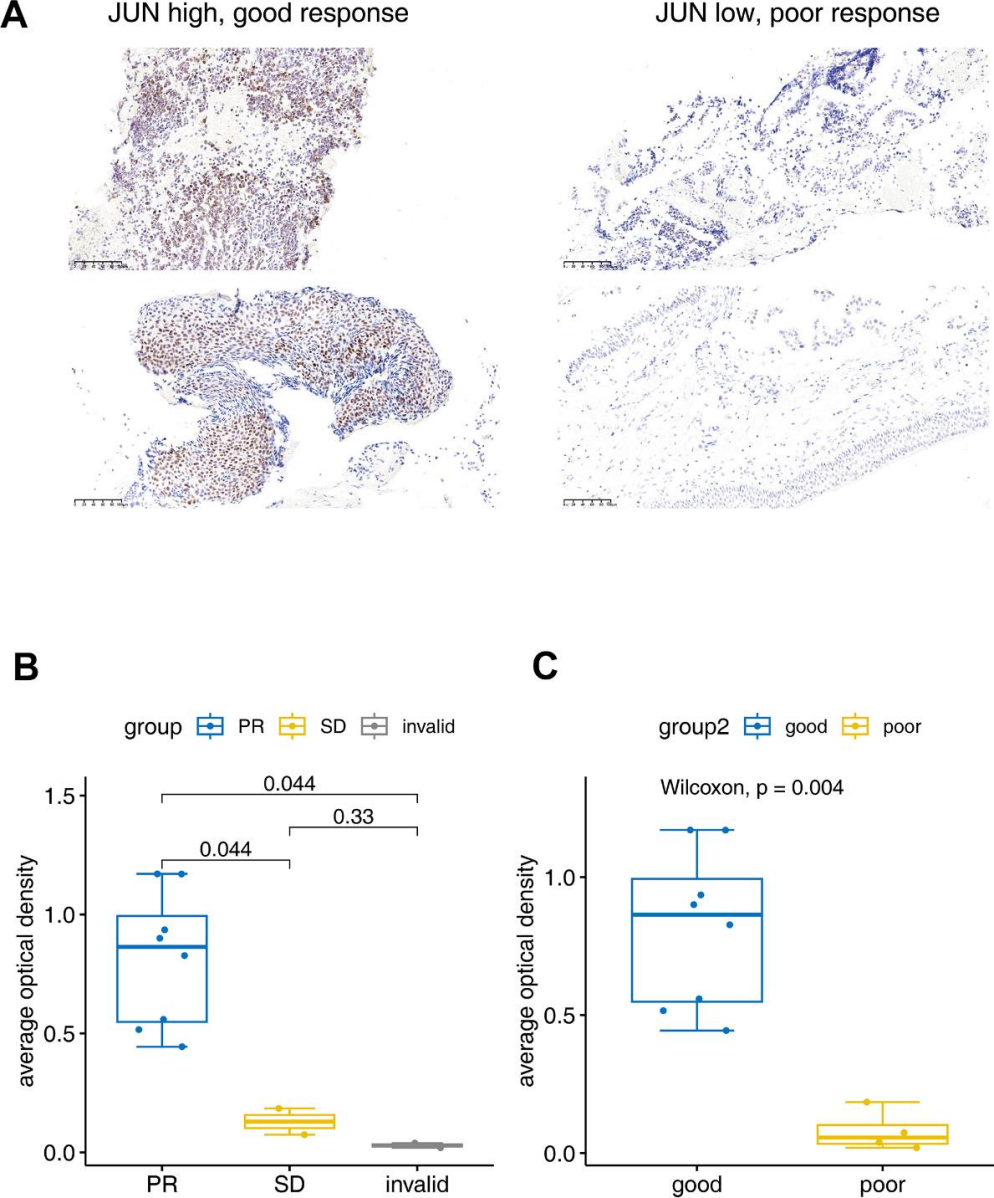
**Immunohistochemical verification of JUN expression.** (**A**) The JUN expression in good and poor response patients for PD1 treatment. (**B**, **C**) Bar plot for the average optical density of immunohistochemical imaging. PR: Partial response (sample number=8), SD: Stable disease (sample number=2), invalid (sample number=2). Good: (sample number=8), poor: (sample number=4). The test was performed with non-parametric Wilcoxon test.

The IHC experiment was performed to further identify the JUN expression in lung cancer tissues. Two invalid, two SD and eight PR samples were enrolled to verify the JUN expression. IHC results showed that the JUN was highly expressed in the good response (PR) tissue, and low expressed in the poor response (SD or invalid) tissue (Figure 6A). Statistical results also showed that the JUN was highly expressed in the PR tissue, and low expressed in the invalid or SD tissue (Figure 6B, 6C). These statistics result also confirmed the findings in the single-cell sequencing analysis.

## DISCUSSION

Here, we applied bioinformatic re-analysis, clustering T lymphocyte cell subpopulations, and RNA sequencing throughout the course of anti-PD-1 treatment to validate the key finding of previous studies. T lymphocytes, as the direct target of PD-1/PD-L1 blockade treatment, are known to be highly heterogeneous on the surface of T cells, and only a subset of T cells in a subpopulation of people are responsive to tumor-related antigens [21]. Recent studies reported that the heterogeneity of immune cells in LUAD tumor tissue can typically be identified using scRNAseq [22–24]. Previous studies on the mechanisms of PD-1 blockade therapy have primarily focused on the bulk-tissue level, where it is difficult to find individual differences that affect the response to PD-1 blockade therapy [5, 25, 26]. Single-cell RNA sequencing technology can be used to investigate cellular and molecular mechanisms at the single-cell level which provides a deeper understanding of the cellular and molecular mechanisms of immunotherapy and can be used to find better potential targets for predicting response, drug action, drug concomitant diagnostic markers, and optimal drug regimens. For example, Zhou et al. found that tumor-induced macrophages and CD8 T lymphocytes in pancreatic tissue-specific resident cells correlated with the response to anti-PD-1 therapy using scRNAseq [27]. This finding suggests that the response of a solid tumor to anti-PD-1 therapy may be correlated with the types and numbers of immune cells, including lymphocytes, macrophages, and tumor cells.

One of the great challenges in cancer immunotherapy research is to elucidate the mechanisms of cancer cell growth, migration, metastasis, and immune tolerance. In our findings, the scRNAseq data of patients with LUAD before and during ongoing treatment with PD-1 blockade revealed that the immune microenvironment of patients with better treatment response included CD4 Treg, non-exhausted CD8 Tnon-ex, CD4 Tcm, CD4 Tnaïve, and CD4 Treg subclusters (Figure 1E, 1G). In addition, the cumulation of CD8 Tnon-ex of Group Bpost was shown to decrease rapidly compared with that of Group Bpre after anti-PD-1 therapy (Figure 3C), suggesting a key role for CD8 Tex cells and precursor cells in PD-1 blockade treatment; this result agrees with those of previous studies [20, 28]. However, due to our selection of enrolled patients (refer to methods), we also found that some interesting phenomena occur, such as abnormal T-cell activation in the poor response to anti-PD-1 treatment (Figure 1F, 1G). To examine these phenomena, we extracted CD4 T cells and CD8 T cells cluster data and performed clustering analysis to determine if there was an association between the JUN gene and PD-1 signaling pathway activation (Figures 2A, 3A). We found that the JUN gene of CD8 T cells in major subpopulations differential expression in the poor response to anti-PD-1 therapy group (Figure 4B, 4D). These findings suggest that CD8 T lymphocyte subclusters may contribute significantly to the progression or regression of LUAD, suggesting a potential predictive role of JUN in CD8 T lymphocytes and subpopulations.

JUN is a proto-oncogene, which is an AP-1 transcription factor subunit that is broadly expressed in normal tissue and immune cells. Diseases associated with JUN include breast cancer and sarcoma. It has participated many keys signaling pathways, including the MyD88-dependent cascade, which is initiated by the intranuclear body and prolactin signaling pathway. To further demonstrate that JUN is associated with PD-1 signaling pathway activation (search in KEGG database), we extracted the RNA sequencing data of lung cancer patients with LUAD from the TCGA database to analyze the correlation between JUN and PD-1 blockade treatment response. We found that the correlation was not high in patients with poor PD-1 treatment response (R=0.034, *P*=0.46, Figure 4E), suggesting that it might be not associated with PD-1 blockade treatment effect in patients. In addition, a survival analysis suggested that patients with high JUN gene expression had a better clinical prognosis (Figure 5D).

To further support this finding, we had tested and analyzed lung adenocarcinoma biopsy tissue samples using mixed tissue RNA sequencing and found that the JUN gene was statistically associated with successful PD-1 blockade therapy (Figure 5C). However, the relative expression of JUN in the good response to PD-1 blockade therapy group was higher than that of the poor response group. This contrary finding may be the result of using bulk-RNA sequencing of tumor tissue, as JUN is also differentially expressed on other cells. In addition, we performed an in-depth analysis of immune cell interactions and found that Treg cells may indirectly affect differential of CD8 sub-clusters and expression JUN gene through signaling pathways, such as PI3K-Akt, TGF-beta, and MAPK, phosphorylating AP1 protein and promoting immune cell proliferation (Figures 2A, 3D), differentiation, and immune response (search in KEGG database). Prior studies have also reported that activated c-JUN Cellular Jun (Cellular JUN) is highly expressed at the invasive front of breast tumors and is closely associated with tumor cell proliferation and angiogenesis [29]. Thus, c-JUN and JUNB (a subtype of JUN) play important roles in lymphoid-resident CD8α-related conventional dendritic cells 1 (cDC1, a subset of conventional dendritic cells), which could affect the diversity, function, and maintenance of cDC1 [30]. Therefore, targeting c-JUN/AP-1 (activating protein-1) may provide new therapeutic approaches for blocking tumor angiogenesis. Finally, we found JUN to cause the activation or inhibition of the PD-1/PDL-1 signaling pathway indirectly, and maybe a potential response predictor for PD-1 blockade treatment.

Although we analyzed the cellular and molecular mechanisms of PD-1 blockade combined with chemotherapy in LUAD patients using scRNAseq, this study has some limitations. For example, we used scRNAseq data shared by previous authors and TCGA database for bioinformatic experimental analysis. Wet experimental aspects were validated using bulk RNA sequencing of samples from clinically enrolled patients, and further *in vivo* and *in vitro* experimental evidences were lacking. The exact mechanism by which JUN is involved in regulating PD-1/PD-L1 signaling pathway activation, and how it affects targeted immunotherapy requires further investigation in animal model.

Importantly, R_O/E_ plot analysis and cellular interaction plots indicated that CD8 T cells and CD4 Treg cells were shown to be heterogeneous subtypes and have unique interactions in post-group compared to that of pre-group (Figure 3D). These findings indicate that the response to PD-1 blockade therapy may correlate with Treg cells and CD8 T cell subpopulations (Figures 2B, 2C, 3C, 3D). The ratios of observed cell numbers to random expectations (Ro/e) method uses cell count and chi square test to calculate the expected and observed coefficients, which can effectively avoid errors caused by sequencing imbalance and observed on the change trend. All results in this article were based on limited single cell RNA sequencing data, which could result in bias and more single-cell data were needed to support the conclusion. The survival analysis of JUN was based on lung adenocarcinoma data from TCGA database, which may mix samples of different subtypes and had a certain impact on the results. For our internal IHC data, due to the small sample size, may also cause bias in the results. Furthermore, the role of JUN discovered in this study in lung adenocarcinoma still needs more experimental validation.

In summary, the results from our study further our understanding of immune cell profiling before and during PD-1 blockade therapy and may provide a valuable predictor of PD-1 treatment response for future clinical research in pharmacogenomics.

## CONCLUSIONS

We identified a new valuable gene, JUN, and described the unique pattern of cellular microenvironment that determines response to PD-1 blockade therapy on lung cancer patient. Furthermore, the expression level on CD8 lyphocytes sub-clusters of JUN may have predictive value in determining the response to PD-1 blockade therapy.

## Supplementary Material

Supplementary Tables
