# Supplementary material for: Identification of JUN gene and cellular microenvironment in response to PD-1 blockade treatment in lung cancer patients via single-cell RNA sequencing
Source: Aging (Albany NY). 2024 Jun 13;16(12):10348–65. doi: 10.18632/aging.205932 (PMC11236306; doi:10.18632/aging.205932)
Supplement: Supplementary Tables [file aging-16-205932-s001.pdf]

SUPPLEMENTARY TABLES

Supplementary Table 1. Clinical characteristics of patients enrolled in this study.

New name	Primary sample name	Patients ID	Age	Gender	Tumor type	Treatment	Response	Treatment Hx	Biopsy site	Timepoint
A01_ut_meta	P010.pre.01	P010	53	Male	LUAD	Pembrolizumab + Carboplatin + Pemetrexed	Yes	Pre-treatment	LN metastasis	A01pre
A02_ut_meta	P019.pre.01	P019	73	Female	LUAD	Pembrolizumab + Carboplatin + Pemetrexed	Yes	Pre-treatment	LN metastasis	A02pre
A01_tr_meta	P010.post.01	P010	53	Male	LUAD	Pembrolizumab + Carboplatin + Pemetrexed	Yes	On treatment	LN metastasis	A01post
A02_tr_meta	P019.post.01	P019	73	Female	LUAD	Pembrolizumab + Carboplatin + Pemetrexed	Yes	On treatment	LN metastasis	A02post
B01_ut_meta	P001.pre.01	P001	57	Female	LUAD	Pembrolizumab + Carboplatin + Pemetrexed	Yes	Pre-treatment	LN metastasis	B01pre
B02_ut_meta	P013.pre.01	P013	61	Female	LUAD	Pembrolizumab + Carboplatin + Pemetrexed	Yes	Pre-treatment	Liver metastasis	B02pre
B02_tr_meta	P001.post.03	P001	57	Female	LUAD	Pembrolizumab + Carboplatin + Pemetrexed	No	On treatment	Right lung tumour	B02post
B02_tr_meta	P013.post.03	P013	61	Female	LUAD	Pembrolizumab + Carboplatin + Pemetrexed	No	On treatment	LN metastasis	B02post
C01_ut_pri	P029.pre.01	P029	52	Male	LUAD	Pembrolizumab + Carboplatin + Pemetrexed	Yes	Pre-treatment	Left lung tumour	C01pre
C02_ut_pri	P030.pre.01	P030	65	Male	LUAD	Pembrolizumab + Carboplatin + Pemetrexed	Yes	Pre-treatment	Right lung tumour	C02pre
C03_ut_pri	P033.pre.01	P033	48	Male	LUAD	Pembrolizumab + Carboplatin + Pemetrexed	Yes	Pre-treatment	Right lung tumour	C03pre
C04_ut_pri	P035.pre.01	P035	71	Male	LUAD	Pembrolizumab + Carboplatin + Pemetrexed	Yes	Pre-treatment	Right lung tumour	C04pre
C01_tr_pri	P029.post.01	P029	52	Male	LUAD	Pembrolizumab + Carboplatin + Pemetrexed	Yes	On treatment	Left lung tumour	C01post
C02_tr_pri	P030.post.01	P030	65	Male	LUAD	Pembrolizumab + Carboplatin + Pemetrexed	Yes	On treatment	Right lung tumour	C02post
C03_tr_pri	P033.post.01	P033	48	Male	LUAD	Pembrolizumab + Carboplatin + Pemetrexed	Yes	On treatment	Right lung tumour	C03post
C04_tr_pri	P035.post.01	P035	71	Male	LUAD	Pembrolizumab + Carboplatin + Pemetrexed	Yes	On treatment	Right lung tumour	C04post

Clinical information from the original text, reorganize and number it. Among them, Pre treatment is defined as Pre for the untreated group, and On treatment is defined as Pos for the treatment group.

Supplementary Table 2. Bulk-RNAseq validation.

Grouped	Response	Patient ID	Hospital ID	Gender	Age	Pathological type (before surgery)	Biopsy
Good Response	MPR	A618_139_T_MPR	161891	male	66	LUAD	right lung
Poor Response	SD	A618_111_TSD	160929	male	64	LUAD	left lung
Poor Response	SD	A618_114_TSD	156260	male	47	LUAD	right lung
Good Response	pCR	A618_88_T_pCR	157558	male	73	LUAD	left lung
Good Response	MPR	A618_59_T_MPR	155967	male	69	LUAD	left lung
Poor Response	SD	A618_101_TSD	152870	male	65	LUAD	left lung
Poor Response	SD	A618_128_TSD	157955	male	45	LUAD	right lung
Poor Response	SD	A618_91_TSD	158496	male	55	LUAD	left lung
Poor Response	SD	A618_106_TSD	157780	male	65	LUAD	right lung
Good Response	MPR	A618_62_T_MPR	157416	male	69	LUAD	right lung
Good Response	pCR	A618_71_T_pCR	154853	male	56	LUAD	right lung
Good Response	pCR	A618_96_T_pCR	158459	male	67	LUAD	right lung
Good Response	pCR	A618_103_T_pCR	159844	male	75	LUAD	left lung
Good Response	MPR	A618_136_T_MPR	157786	male	55	LUAD	right lung
Poor Response	SD	A618_47_TSD	152297	female	65	LUAD	left lung
Poor Response	SD	A618_125_TSD	156386	female	72	LUAD	right lung
Poor Response	SD	A618_44_TSD	152560	male	56	LUAD	right lung

Bulk RNA-seq data analysis, in which PCR (complete pathological response) and MPR (significant pathological response) are defined as good responses. SD (stable disease), PD (progressive disease) are defined as adverse reactions.

Supplementary Table 3. IHC validation.

Pathology number	SEX	Age	Prognosis
B2101862	female	55	PR
B2108744	male	75	PR
B2113067	male	76	SD
B2100531	male	63	PR
B2006179	male	66	PR
B2001556	female	50	SD
B2002417	male	53	invalid
B2104352	male	71	PR
B2202174	male	72	PR
B2007434	male	72	PR
B2004459	male	40	PR
B2203394	male	74	PR
B2215850	female	61	invalid
B1810548	male	58	PR
B2202570	male	65	PR
B2206675	female	70	SD
B2101407	male	57	PR
B2106387	male	65	PR

18 case slices from Tongren Hospital were subjected to IHC histochemical staining experiments, PR (pathological response). SD (stable disease), invalid (invalid group).
